# Trauma-related altered states of consciousness in post-traumatic stress disorder patients with or without comorbid dissociative disorders

**DOI:** 10.1080/20008198.2018.1544025

**Published:** 2018-11-14

**Authors:** Harald Bækkelund, Paul Frewen, Ruth Lanius, Akiah Ottesen Berg, Espen Ajo Arnevik

**Affiliations:** a Research Institute, Modum Bad, Vikersund, Norway; b Norwegian Center for Violence and Traumatic Stress Studies, Oslo, Norway; c Department of Psychiatry, Western University, London, Canada; d Norwegian Centre for Mental Disorders Research (NORMENT), K. G. Jebsen Centre for Psychosis Research, Institute of Clinical Medicine, University of Oslo, and Division of Mental Health and Addiction, Oslo University Hospital, Oslo, Norway; e National Advisory Unit on Substance Use Disorder Treatment, Oslo University Hospital, Oslo, Norway

**Keywords:** Dissociation, post-traumatic stress disorder, dissociative disorders, trauma-related altered states of consciousness, child abuse and neglect, 4-D model, disociación, trastorno de estrés postraumático, trastornos disociativos, estados alterados de conciencia relacionados al trauma, maltrato infantil y negligencia, modelo 4-D, 解离, 创伤后应激障碍, 解离障碍, 创伤相关的意识状态改变, 虐待和忽视儿童, 4-D模型, • The ‘4-D model’ of dissociation categorizes symptoms into trauma-related altered states of consciousness (TRASC) and normal waking consciousness (NWC), which occur along four dimensions: time, thought, body and emotion. •The main predictions of the model were supported in patients with PTSD, with and without comorbid dissociative disorders. •The 4-D model represents a promising framework for understanding dissociation across trauma-related disorders.

## Abstract

**Background**: The four-dimensional (‘4-D’) model has been proposed as a theoretical framework to understand and delineate trauma-related dissociative experiences, categorizing symptoms into trauma-related altered states of consciousness (TRASC) and normal waking consciousness (NWC), which occur along four dimensions: time, thought, body and emotion.

**Objective**: The main aim of the present study was to evaluate the validity of this model in patients with post-traumatic stress disorder (PTSD), with and without comorbid dissociative disorders.

**Method**: The predictions of the 4-D model were tested in 142 patients with PTSD, with (*N* = 46) and without (*N* = 96) comorbid dissociative disorders.

**Results**: As predicted by the 4-D model, experiences of TRASC were less frequent and more specifically related to other measures of dissociation, dissociative disorder comorbidity and a history of childhood sexual abuse compared to experiences of NWC. The predicted lower intercorrelation of TRASC was not supported.

**Conclusion**: The 4-D model represents a promising framework for understanding dissociation across trauma-related disorders.

## Abstract (es)

**Antecedentes**: El ‘modelo 4-D’ ha sido propuesto como un marco teórico para entender y delinear las experiencias disociativas relacionadas al trauma, categorizando los síntomas en estados alterados de conciencia relacionados al trauma (TRASC en su sigla en inglés) y la conciencia en vigilia normal (NWC en sus siglas en inglés), que ocurren a lo largo de cuatro dimensiones: (1) tiempo; (2) pensamiento; (3) cuerpo; y (4) emoción. El principal objetivo del presente estudio fue evaluar la validez de este modelo en pacientes con Trastorno de Estrés Postraumático (TEPT), con y sin trastornos disociativos comórbidos.

**Métodos**: Los predictores del modelo 4-D fueron probados en 142 pacientes con TEPT, con (*N*=46) y sin (*N*=96) trastornos disociativos comórbidos.

**Resultados**: Como predice el modelo 4-D, las experiencias de los TRASC fueron menos frecuentes y más específicamente relacionados a otras medidas de disociación, comorbilidad del trastorno disociativo y una historia de abuso sexual infantil en comparación a las experiencias de NWC. La predicción de la intercorrelación más baja de los TRASC no fue confirmada.

**Conclusión**: El modelo 4-D representa un prometedor marco para el entendimiento de la disociación de forma transversal en los trastornos relacionados al trauma.

## Abstract (zh)

背景：‘4-D模型’已被提议作为理解和描述创伤相关的解离经验的理论框架，将症状分类为创伤相关的意识改变状态（TRASC）和正常清醒意识（NWC），其中有四个维度：（1）时间; （2）思想; （3）身体; （4）情绪。本研究的主要目的是评估该模型在合并或者未合并的解离障碍的创伤后应激障碍（PTSD）患者中的有效性。

方法： 对142例PTSD患者考察4-D模型的预测性，其中N = 46共病解离障碍，*N* = 96无共病。

结果： 正如4-D模型预测的那样，与NWC的经验相比，TRASC的经验较少出现，并且更具体地与其他解离症状、解离障碍共病证和儿童性虐待史相关。预期的TRASC的内部相关性没有得到支持。

结论： 4-D模型代表了一个理解创伤相关疾病中的解离症状的框架。

## 1. Introduction

The fifth edition of the Diagnostic and Statistic Manual of Mental Disorders (DSM-5) (American Psychiatric Association, 2013) recognizes a ‘close relationship’ (p. 291) between dissociative disorders and trauma- and stressor-related disorders, such as post-traumatic stress disorder (PTSD). This relationship manifests in both symptomatic overlap and high comorbidity. Patients with PTSD generally experience increased levels of dissociative symptoms compared to trauma-affected people without PTSD (Carlson, Dalenberg, & McDade-Montez, 2012; Dorahy & van der Hart, 2015), while a dissociative subtype of PTSD patients (DPTSD) is characterized by pervasive symptoms of derealization and depersonalization and often related to childhood abuse (Lanius, Brand, Vermetten, Frewen, & Spiegel, 2012; Lanius et al., 2010). In addition, PTSD symptoms such as flashbacks and trauma-related amnesia are regarded by many theorists as dissociative phenomena (Dell, 2006b; Frewen & Lanius, 2015; Nijenhuis, Hart, & Steele, 2010). Patients with dissociative disorders, which are characterized by pervasive and severe dissociative symptoms, almost invariably also suffer from PTSD, with comorbidity rates from 88% to 97% (Foote, Smolin, Neft, & Lipschitz, 2008; Rodewald, Wilhelm-Göling, Emrich, Reddemann, & Gast, 2011).

However, several authors have noted that confusion remains as to how dissociation should be defined and which symptoms and experiences should be included as dissociative (Brown, 2006; Dell, 2009; Holmes et al., 2005). Empirical data clearly suggest that dissociative experiences do not form a unitary dimension, but are better understood as multidimensional phenomena (Briere, Weathers, & Runtz, 2005; Bryant, 2007; Dell, 2006b; Holmes et al., 2005). It is, for instance, common to differentiate between compartmentalization, involving an inability to access or control normal mental processes, and detachment, such as depersonalization, derealization and numbing. For research, theory and clinical practice it is therefore critical that researchers define clearly the specific phenomenological construct of dissociation to which they are referring (e.g. dissociative flashbacks vs depersonalization vs derealization vs identity dissociation) (Brand & Frewen, 2017).

To better understand and differentiate dissociative phenomena, Frewen and Lanius (2015) argue that it is necessary to distinguish between subtypes of trauma-related reactions that are intrinsically dissociative and those that are not intrinsically dissociative in nature but rather may present as forms of distress within normal waking consciousness (NWC). The four-dimensional (4-D) model emphasizes phenomenological first-person descriptions of conscious experience and how such experiences might be affected by trauma. The more inherently dissociative reactions, involving a distinct non-self-referential form of processing (i.e. ‘this isn’t me’, ‘this is happening to somebody else’) are labelled trauma-related altered states of consciousness (TRASC). Specifically, four phenomenological dimensions of a person’s awareness that can be affected during as well as in the aftermath of trauma are described: time, thought, body and emotion. TRASC of the time dimension was proposed to involve traumatic flashbacks that involve a profound sense of reliving as compared to the NWC experience of upsetting and intrusive memories that do not exhibit a reliving component. Negative voice-hearing is characterized as a TRASC phenomenon of the thought dimension, whereas NWC experiences involve negative self-referential cognitions occurring in first-person perspective. Along the body dimension, disembodied experiences (e.g. depersonalization) are regarded as TRASC, while embodied forms of distress (e.g. hyperarousal) are considered as a symptom of NWC. Lastly, severe emotional numbing and disowned/compartmentalized emotions are proposed to be TRASC, while NWC expressions will involve generally negative affects (e.g. sadness, anger and shame) that are not disowned/compartmentalized. The 4-D model contends that symptoms of TRASC can be experienced both together with and apart from identity alterations that occur in severe dissociative disorders, such as dissociative identity disorder (DID). As such, experiences of TRASC are not specific for dissociative disorders, but are thought to represent transdiagnostic states across trauma-related disorders.

To investigate the validity of the 4-D model, Frewen and Lanius (2015) make four testable predictions. First, because experiences of NWC distress are conceptualized as being within the typical and normal state of humans, they should be more common and frequently endorsed than experiences of TRASC. Secondly, because experiences of TRASC are hypothesized to be more compartmentalized as dissociative experiences, they are hypothesized to less intercorrelated (Brown, 2006; Holmes et al., 2005) across the four dimensions than in the form of NWC distress, especially when measured over time (i.e. dimensions of NWC should correlate more strongly than dimensions of TRASC). Thirdly, the 4-D model predicts that experiences of TRASC should be more frequently reported by people with high scores on other measures of dissociation, especially other measures of pathological dissociation. Finally, Frewen and Lanius (2015) hypothesize that TRASC typically develop as a result of repeated traumatization at sensitive periods of development, and therefore predict that experiences of TRASC will be related more specifically than NWC distress to a history of repeated traumatization and childhood abuse.

Several studies have investigated and largely found support for the predictions of the 4-D model in different samples, such as undergraduate students (Brown & Frewen, 2017; Frewen & Lanius, 2014a), clinical populations with and without PTSD (Frewen, Kleindienst, Lanius, & Schmahl, 2014; Frewen & Lanius, 2014a), acutely traumatized individuals (Frewen et al., 2015) and community samples recruited online (Frewen, Brown, & Lanius, 2017; Tzannidakis & Frewen, 2015). The first prediction, that NWC will be endorsed more frequently than TRASC, and the third prediction, that TRASC will be more strongly associated with other measures of dissociation, have been supported across all studies to date. The second prediction, hypothesizing higher intercorrelations among the dimensions in NWC distress than the TRASC form, was supported in all studies except in a sample of 258 women diagnosed with borderline personality disorder, with and without comorbid PTSD (Frewen et al., 2014). Finally, support for the fourth prediction, that symptoms of TRASC will be specifically related to a history of repeated traumatization and childhood abuse, has been less consistent. Frewen and Lanius (2014a) found an association between a history of childhood sexual abuse (CSA) and voice-hearing in women with PTSD, but no general relationship between experiences of TRASC and other forms of childhood abuse was demonstrated. In comparison, Frewen et al. (2014) found a specific relationship of TRASC with emotional neglect, but not across other forms of childhood trauma. Other investigations have generally found positive correlations between reports of childhood trauma and both NWC distress and TRASC, but with no consistent pattern regarding specificity emerging.

Although the studies described above largely provide support for the 4-D model of dissociation, an important goal of the theory is to describe dissociation transdiagnostically across the spectrum of trauma-related disorders. Therefore, a major limitation of the research to date is that, to our knowledge, the 4-D model has never been investigated in a sample of patients with diagnosed dissociative disorders. The main aim of the present study was therefore to evaluate the validity of the 4-D model of trauma-related dissociation by testing predictions in patients with histories of childhood abuse and diagnostic presentations including PTSD and dissociative disorders.

The hypotheses are:

1. Experiences of TRASC will be specifically related to a self-reported history of childhood abuse.
2. Comorbid dissociative disorder will be specifically associated with higher endorsement of experiences of TRASC, not NWC distress.
3. Experiences of TRASC will be less frequently endorsed than distress associated with NWC in PTSD patients with and without a comorbid dissociative disorder.
4. Experiences of TRASC will be less intercorrelated than experiences of NWC.
5. Experiences of TRASC will be more strongly related to other measures of dissociation than experiences of NWC.

## 2. Methods

### 2.1. Participants and procedures

All participants were recruited from patients referred to an outpatient clinic specializing in the treatment of trauma-related disorders, as part of an ongoing clinical trial (Clinical Trials NCT02450617). The study was approved by the Norwegian Regional Committees for Medical and Health Research Ethics (REC) and funded by Modum Bad Psychiatric Hospital. Subjects aged between 18 and 65 years who had sufficient competence in Norwegian and reported childhood abuse and trauma-related symptoms were eligible for inclusion. Exclusion criteria included acute suicidality, severe substance abuse, psychotic illness, current life crisis (e.g. ongoing abuse, divorce, court case, somatic disease in spouse or children), neurological illness, intellectual disability and life-threatening somatic disease.

Participants (*N* = 177) were enrolled and completed a comprehensive diagnostic assessment by trained interviewers. Only patients who fulfilled criteria for PTSD were included in the present study, as this was a requirement for the clinical trial. One patient with a dissociative disorder failed to meet criteria for PTSD. Also, 34 patients were excluded as they had not filled out the measure used to operationalize 4-D scores. Analysis of available data found that excluded patients did not differ from the included patients on age, gender or symptomatology assessed by other measures. The resulting sample (*N* = 142) consisted of 46 patients with a comorbid dissociative disorder and 96 without such comorbidity. Of the patients with dissociative disorders, 25 fulfilled criteria for DID (characterized by pronounced dissociative amnesia and the presence of two or more distinct personality states), and 21 fulfilled criteria for other specified dissociative disorders (characterized by chronic dissociative symptoms, but with less distinct identity parts as in DID). These two disorders are collectively categorized as complex dissociative disorders (CDDs) (Dell, 2009). The vast majority of the sample were female (88.7%) and the mean age was 39.1 years (SD = 10.09).

### 2.2. Measures

All assessments used approved Norwegian translations. Patients could request assistance if they had difficulty understanding the questions or information.

The *Post-traumatic Symptom Scale – Interview* (Foa, Riggs, Dancu, & Rothbaum, 1993) was used to assess PTSD. Because of a lack of validated diagnostic instruments for DSM-5 at the start of data collection, diagnosis was based on DSM, 4th Edition (DSM-IV) criteria and did not assess DPTSD.

The *Structured Clinical Interview for DSM-IV Dissociative Disorders* (Steinberg, Cicchetti, Buchanan, & Hall, 1993) was used to assess dissociative disorders.

The *Mini-International Neuropsychiatric Interview* (MINI) (Sheehan et al., 1998) and *Structured Clinical Interview for DSM-IV Axis II Personality Disorders* (SCID-II) (First, Benjamin, Gibbon, Spitzer, & Williams, 1997) were administered to assess general psychopathology and personality disorders.

Background information and sociodemographic data were registered using a generic form.

The *Multidimensional Inventory of Dissociation* (MID) (Dell, 2006a) is a self-report measure with 218 items that uses a 11-point Likert scale. Of these items, 168 measure different dimensions of dissociation, while 50 measure validity. The measure was translated into Norwegian and back-translated into English. The back-translation was checked for inconsistencies and approved by the original author. The MID was used in this study to quantify experiences of NWC distress in comparison to TRASC, as described further in Section 2.3.

The *Childhood Trauma Questionnaire – Short Form* (CTQ-SF) (Bernstein et al., 1994; Dovran et al., 2013) is an extensively used measure for retrospective reporting of experiences of abuse and neglect in the respondents’ childhood. CTQ-SF has 28 items scored from 0 (‘never true’) to 5 (‘very often true’). Cronbach’s alpha for CTQ total score in the present sample was .83, indicating good internal consistency. CTQ has five subscales: emotional neglect (Cronbach’s *α* = .82), physical neglect (Cronbach’s *α* = .64), emotional abuse (Cronbach’s *α* = .93), sexual abuse (Cronbach’s *α* = .79) and physical abuse (Cronbach’s *α* = .85).

The *Dissociative Experiences Scale* (DES) (Bernstein & Putnam, 1986; Carlson & Putnam, 1993) is a 28-item self-report questionnaire designed to measure both pathological and non-pathological dissociative experiences. It uses an 11-point Likert scale, asking respondents to indicate the percentage of their time the experiences affect them. A meta-analysis reported an overall Cronbach’s alpha coefficient of .96 and a significant difference in DES scores between dissociative patients and non-dissociative controls (Van Ijzendoorn & Schuengel, 1996). Cronbach’s alpha in the present sample was also .96.

The *Somatoform Dissociation Questionnaire* (SDQ) (Nijenhuis, Spinhoven, van Dyck, van der Hart, & Vanderlinden, 1996) has 20 items asking about somatoform dissociative symptoms on a five-point scale. SDQ-20 has shown good reliability and validity (Nijenhuis, Spinhoven, van Dyck, van der Hart, & Vanderlinden, 1998). Cronbach’s alpha in the present sample was .88.

The *Symptom Checklist-90 Item – Revised* (SCL-90-R) (Derogatis, 1994) is a widely used self-report measure for psychological symptoms and distress consisting of 90 items. The summary score, referred to as a Global Severity Index (GSI), is often used as a measure of general psychopathology. Cronbach’s alpha in the present sample was .97.

### 2.3. Data reduction

The TRASC and NWC factors for each dimension were calculated using items from the MID (Table 1). The originators of the 4-D model (RL and PF) selected items thought to best correspond to NWC and TRASC on each phenomenological dimension, while being blind to the data. The selections were compared to selections of MID items used in previous work (Frewen & Lanius, 2015), and the final items were decided unanimously. Mean scores on the selected items were used to give each participant a score for NWC and TRASC on each dimension (i.e. eight different scores; Table 1).

**Table 1.** Items from the Multidimensional Inventory of Dissociation (MID) descriptions of trauma-related altered states of consciousness (TRASC) and normal waking consciousness (NWC) across the four-dimensional (4-D) model dimensions of time, thought, body and emotion.

	TRASC	NWC
Time	Items 14, 114, 145 and 146 Cronbach’s *α* = .688 Example: ‘Reliving a past trauma so vividly that you see it, hear it, feel it, smell it, etc.’	Item 115 ‘Bad memories coming into your mind and you can’t get rid of them.’
Thought	Items 6, 30, 42, 61, 84, 118, 140, 159, 171, 199 and 207 Cronbach’s *α* = .936 Example: ‘Hearing a voice in your head that tries to tell you what to do.’	Items 22 and 151 Cronbach’s *α* = .696 Example: ‘Strong thoughts in your head that “come from out of nowhere”.’
Body	Items 3, 91, 126 164, 172, 191, 197 and 203 Cronbach’s *α* = .889 Example: ‘Standing outside of your body, watching yourself as if you were another person.’	Item 125 ‘Re-experiencing *body sensations* from a past traumatic event.’
Emotion	Items 27, 60, 169 and 196 Cronbach’s *α* = .688 Example: ‘Very strong feelings (e.g. fear, or anger, or emotional pain and hurt) that *suddenly go away*.’	Items 32, 57 and 185 Cronbach’s *α* = .743 Example: ‘Your mood changing rapidly without any reason.’

### 2.4. Statistical analysis

All statistical analyses were carried out using SPSS version 25. Analyses were performed as outlined in previous methodology (Brown & Frewen, 2017; Frewen et al., 2014; Frewen & Lanius, 2014b). The first hypothesis regarding the relationship between TRASC/NWC and childhood abuse was investigated by examining bivariate correlations. For the second hypothesis, logistical regressions were performed with CDD comorbidity as a dichotomous outcome and TRASC/NWC variables as predictors, while age and general psychopathology were included as nuisance parameters. To investigate the third hypothesis, we performed within-subjects analyses of variance, with dimension (four levels: time, thought, body and emotion) and form of consciousness (two levels: NWC and TRASC) as factors. Significant results were followed by post-hoc comparisons, using Bonferroni-corrected levels of significance. The fourth hypothesis was tested by calculating intercorrelations between TRASC/NWC scores across dimensions, and comparing these through conversion to *z*-scores (Fisher transformation). Finally, for the fifth hypothesis we performed hierarchical regressions with other measures of dissociation as dependent variables and alternating stepwise order of TRASC and NWC as predictors. Since MID items were used to calculate TRASC/NWC scores, DES and SDQ were used as dependent variables.

## 3. Results

### 3.1. Sample characteristics

Descriptive statistics and group differences between patients with and without comorbid CDD are reported in Table 2. The CDD group was more likely to report a history of inpatient treatment and comorbid psychotic symptoms, as assessed by MINI, and general psychopathology, as assessed by SCL-90. A strong association was found between severe suicidality scores on MINI and CDD comorbidity. Few differences were observed in the level of reported childhood abuse, except for experiences of emotional neglect. The group with comorbid CDD was also significantly younger, by a mean of 4.4 years (SD = 10.36).

**Table 2.** Sample characteristics and clinical comorbidity.

	PTSD with CDD^a^ (*N* = 46)	PTSD without CDD^a^ (*N* = 96)	Difference between groups
Age (years)	36.1 (10.4)	40.5 (9.7)	2.442(138), *p* = .016*
Gender, male	4.3%	14.6%	*χ^2^* = 3.259, *p* = .71
Married or partner	52.4%	43.9%	*χ^2^* = 0.802, *p* = .371
College-level education	46.3%	45.7%	*χ^2^* = 0.005, *p* = .945
GSI	1.95 (0.661)	1.66 (0.664)	*t*(118) = −2.252, *p* = .026*
MINI number of comorbid axis-I disorders	5.2 (2.5)	4.5 (2.3)	*t*(135) = −1.783, *p* = .077
MINI any depressive disorder (present or lifetime)	90.7%	91.5%	*χ^2^* = 0.023, *p* = .879
MINI any bipolar disorder (present or lifetime)	23.3%	19.1%	*χ^2^* = 0.306, *p* = .580
MINI severe suicidality (scored above 2)	72.1%	20.2%	*χ^2^* = 34.26, *p* = < .001**
MINI any anxiety disorder (present or lifetime)	88.4%	85.1%	*χ^2^* = 0.263, *p* = .608
MINI substance abuse	11.6%	13.8%	*χ^2^* = 0.125, *p* = .723
MINI any psychotic disorder (present or lifetime)	30.2%	13.8%	*χ^2^* = 5.162, *p* = .0023*
MINI any eating disorder	18.6%	8.5%	*χ^2^* = 2.914, *p* = .088
SCID-II number of comorbid axis-II disorders	.907 (1.06)	.904 (1.06)	*t*(135) = −.14, *p* = .702
SCID-II borderline personality disorder	14%	12.8%	*χ^2^* = 0.035, *p* = .849
Inpatient treatment ever	74.3%	46.5%	*χ^2^* = 7.345, *p* = .007*
Inpatient treatment last year	35.3%	18.3%	*χ^2^* = 3.656, *p* = .056
Work incapacity	80.5%	75%	*χ^2^* = 0.459, *p* = .498
CTQ total	77.29 (20.235)	71.15 (18.057)	*t*(120) = −1.704, *p* = .091
CTQ – Emotional abuse	17.85 (5.332)	17.63 (5.458)	*t*(120) = −0.216, *p* = .830
CTQ – Physical abuse	12.05 (6.253)	10.40 (5.647)	*t*(120) = −1.473, *p* = .143
CTQ – Sexual abuse	16.17 (7.11)	14.47 (7.27)	*t*(119) = −1.223, *p* = .224
CTQ – Emotional neglect	19.49 (4.879)	17.44 (5.153)	*t*(120) = −2.106, *p* = .037
CTQ – Physical neglect	11.73 (5.301)	11.38 (4.931)	*t*(120) = −0.360, *p* = .719
MID mean score	42.13 (17.486)	21.74 (15.318)	*t*(140) = −7.083, *p* < .001**
SDQ-20	40.667 (12.249)	30.900 (9.057)	*t*(101) = −4.545, *p* < .001**
DES total	42.785 (19.822)	18.912 (16.387)	*t*(103) = −6.519, *p* < .001**

^a^Data are shown as mean (SD) or percentages. PTSD, post-traumatic stress disorder; CDD, complex dissociative disorder; GSI, Global Severity Index; MINI, Mini-International Neuropsychiatric Interview; SCID-II, Structured Clinical Interview for DSM-IV Axis II Personality Disorders; CTQ, Childhood Trauma Questionnaire; MID, Multidimensional Inventory of Dissociation; SDQ, Somatoform Dissociation Questionnaire; DES, Dissociative Experiences Scale. *p < .05, **p < .01.

### 3.2. Hypotheses

Hypothesis 1. Experiences of TRASC will be specifically related to a self-reported history of childhood abuse

Examining bivariate correlations between TRASC/NWC and CTQ scores, mean NWC symptoms were not significantly related to self-reported childhood abuse, while mean TRASC symptoms were correlated with CTQ total score (*r* = .253, *p* = .005) and CTQ sexual abuse (*r* = .295, *p* = .001). Further investigating correlations between CTQ subscales and TRASC/NWC scores across dimensions (Table 3), a significant association between sexual abuse and symptoms of TRASC was replicated across all dimensions. Only NWC symptoms of the body dimension were significantly correlated with any CTQ subscales.

Hypothesis 2. Comorbid dissociative disorder will be specifically associated with higher endorsement of experiences of TRASC, not NWC distress

**Table 3.** Correlations between dimensions of trauma-related altered states of consciousness (TRASC) or normal waking consciousness (NWC), and measures of dissociation and childhood abuse.

	DES	SDQ	CTQ – total	EA	PA	SA	EN	PN
TRASC – Time	.731**	.678**	.178*	.127	.165	.271**	−.017	.006
NWC – Time	.566**	.338**	.127	.157	.015	.099	.121	.042
TRASC – Thought	.662**	.608**	.139	.094	.091	.237**	.034	−.075
NWC – Thought	.515**	.409**	−.047	−.052	−.049	.054	−.100	−.057
TRASC – Body	.777**	.787**	.230*	.146	.162	.280**	.072	.095
NWC – Body	.548**	.447**	.267**	.208*	.169	.244**	.140	.115
TRASC – Emotion	.801**	.718**	.327**	.225*	.269**	.213*	.189*	.182*
NWC – Emotion	.546**	.364**	.057	.033	.047	.108	.043	−.075

DES, Dissociative Experiences Scale; SDQ, Somatoform Dissociation Questionnaire; CTQ, Childhood Trauma Questionnaire; EA, emotional abuse; PA, physical abuse; SA, sexual abuse; EN, emotional neglect; PN, physical neglect. All forms of childhood abuse were measured with the CTQ.*p < .05, **p < .01.

**Figure 1.**
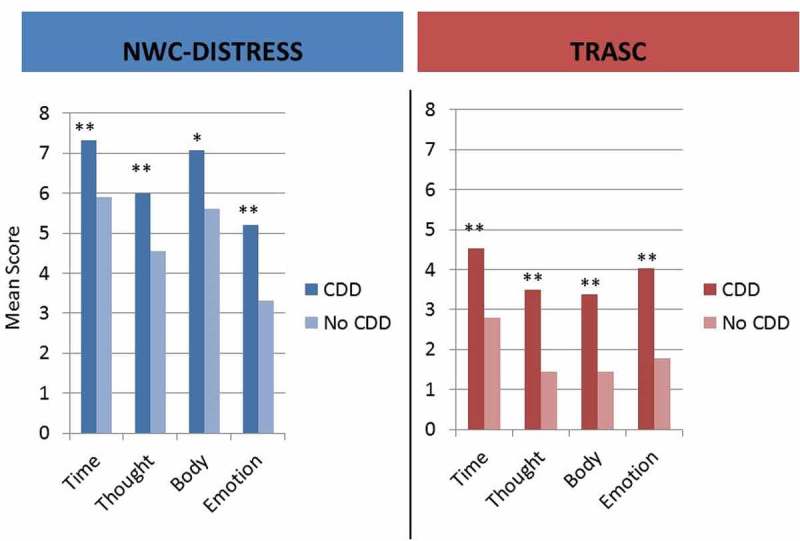
Mean symptom endorsement for each dimension of trauma-related altered states of consciousness (TRASC) and normal waking consciousness (NWC) compared in patients with and without complex dissociative disorder (CDD).*p < .05, **p < .01.

Patients with comorbid CDD endorsed both NWC and TRASC symptoms to a greater degree than patients without comorbid CDD across all four dimensions (Figure 1). Results from logistic regression analyses are shown in Table 4. Consistent with predictions, TRASC symptoms significantly predicted CDD comorbidity across all four dimensions, while controlling for group differences in age and general psychopathology. Scores on NWC distress were not significant as predictors.

Hypothesis 3. Experiences of TRASC will be less frequently endorsed than distress associated with NWC

**Table 4.** Logistic regressions predicting a comorbidity of complex dissociative disorder.

Dimension		*β* (SE)	OR	Model *χ^2^* (df)	Model *r*^2^ (Nagelkerke)
Mean				22.902 (114)**	.250
	TRASC	.758 (.218)**	2.133		
	NWC	−.042 (.157)	0.959		
Time				6.360 (112)*	.075
	TRASC	.233 (.112)*	1.263		
	NWC	.039 (.094)	1.040		
Thought				12.091 (114)**	.138
	TRASC	.353 (.115)**	1.424		
	NWC	.010 (.094)	1.010		
Body				14.284 (111)**	.165
	TRASC	.393 (.125)**	1.482		
	NWC	.022 (.078)	1.022		
Emotion				25.419 (114)**	.275
	TRASC	.526 (.145)**	1.446		
	NWC	.144 (.100)	1.155		

TRASC, trauma-related altered states of consciousness; NWC, normal waking consciousness. Age and Global Severity Index were included as parameters. *p < .05, **p < .01.

Mean symptom endorsement for each dimension of TRASC and NWC compared in patients with and without CDD can be seen in Figure 1. Within the whole sample, both form of consciousness [*F*(1,135) = 397.987, *p* < . 001, *η*^2^ = .747] and dimension of consciousness [*F*(3,405) = 37.004, *p* < .001, *η*^2^ = .215] emerged with significant main effects, with a form Χ dimension interaction [*F*(3,405) = 82.598, *p* < .001, *η*^2^ = .169]. This indicates that although form of consciousness (NWC vs TRASC) predicts endorsement with a large effect size, this association varies across dimensions. Follow-up paired *t*-tests comparing NWC and TRASC on all dimensions were all significant at the < .001 level, with effect sizes ranging from 0.544 to 1.143 in the predicted direction (NWC > TRASC). Performing the same analyses in subsamples with and without comorbid CDD largely replicated these results (see supplementary material).

Hypothesis 4. Experiences of TRASC will be less intercorrelated than experiences of NWC

**Figure 2.**
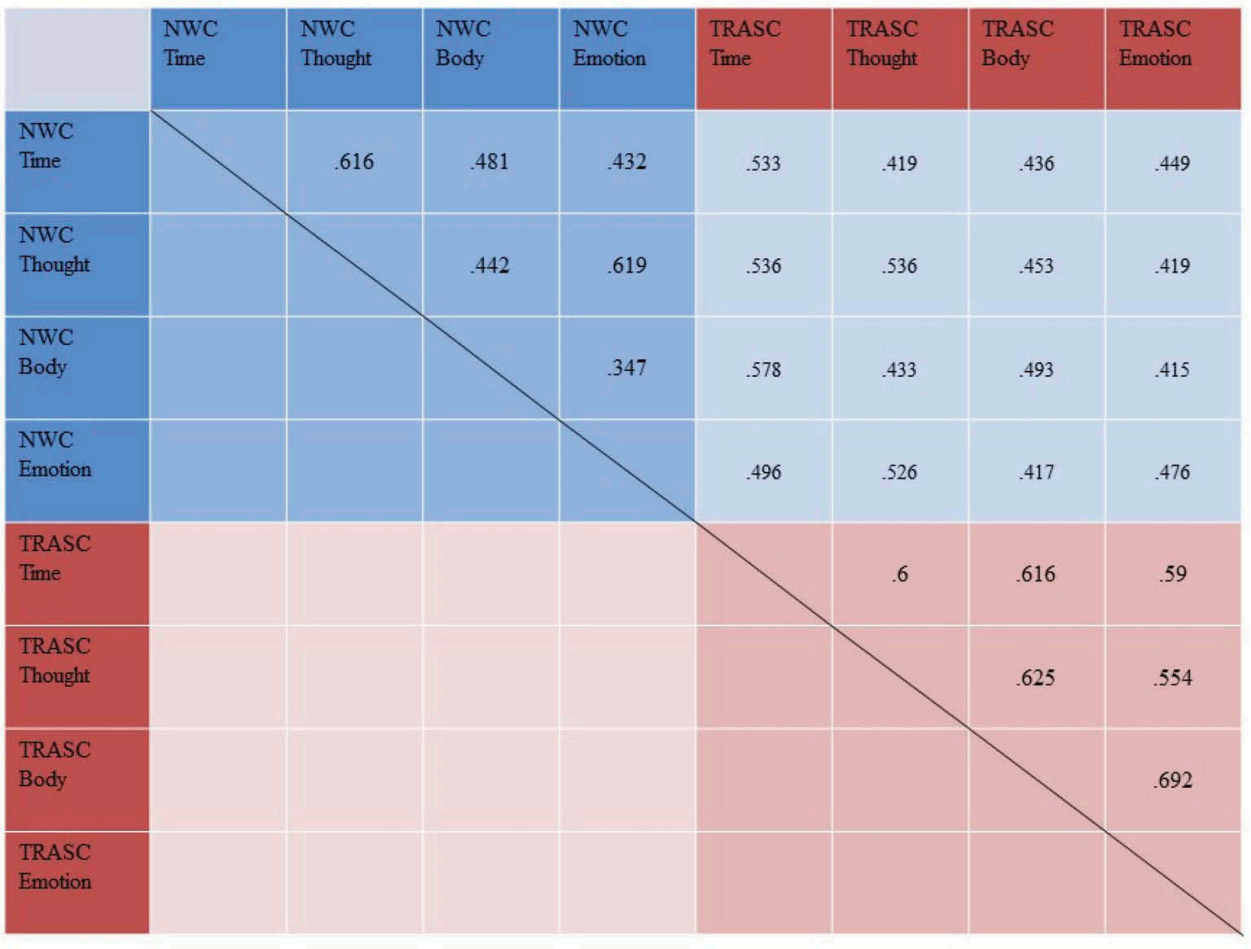
Intercorrelations between the four different dimensions of trauma-related altered states of consciousness (TRASC) and normal waking consciousness (NWC). All correlations are significant at *p *< .001.

Intercorrelations between the four different dimensions of TRASC and NWC can be seen in Figure 2. Contrary to predictions, TRASC subscales generally had higher intercorrelations (M*r* = .612, SD*r* = .046) than NWC subscales (M*r* = .489, SD*r* = .108). In particular, the differential strength of correlation coefficients was found to be statistically significant in three out of six cases, specifically, in the case of the time and emotion dimensions (TRASC *r* = .590, NWC *r* = .432, Δ*r* = .158, *p* = .04), thought and body dimensions (TRASC *r* = .625, NWC *r* = .442, Δ*r* = .183, *p* = .02) and body and emotion dimensions (TRASC *r* = .692, NWC *r* = .347, Δ*r* = .345, *p* < .01). However, the difference was not significant in the case of the time and thought dimensions (TRASC *r* = .600, NWC *r* = .616, Δ*r* = .016, *p* = .81), time and body dimensions (TRASC *r* = .616, NWC *r* = .481, Δ*r* = .135, *p* = .07) or thought and emotion dimensions (TRASC *r* = .544, NWC *r* = .619, Δr = .075, *p* = .29). Moreover, this general, overall trend was replicated in the subsamples with CDD (TRASC M*r* = .567, SD*r* = .126; NWC M*r* = .378, SD*r* = .102) and without comorbid CDD (TRASC M*r* = .529, SD*r* = .061, vs NWC M*r* = .488, SD*r* = .145).

Hypothesis 5. Experiences of TRASC will be more strongly related to other measures of dissociation than experiences of NWC

As reported in Table 3, both NWC and TRASC across all dimensions were significantly correlated with other measures of dissociation. NWC distress failed to significantly incrementally predict scores on DES beyond TRASC scores (Δ*R*^2^ = .009, total *R*^2^ = .763, Δ*F* change = 3.681, *p* = .058), but slightly improved the model for SDQ (Δ*R*^2^ = .018, total *R*^2^ = .684, Δ*F* change = 5.742, *p* = .018). By contrast, symptoms of TRASC incrementally improved predictions for DES (Δ*R*^2^ = .273, Δ*F* = 117.57, *p* < .001) and SDQ (Δ*R*^2^ = .428, Δ*F* = 135.35, *p* < .001) beyond symptoms of NWC, supporting the hypothesis. These results were largely replicated in both subsamples when analysed separately (see supplementary material).

## 4. Discussion

The results of this study found general support for the predictions of the 4-D model of dissociation (Frewen & Lanius, 2015). The theoretical distinction between reactions that are inherently dissociative (TRASC) and those that may remain part of normal waking consciousness (NWC) revealed predicted endorsement rates and correlations with other measures of dissociation across the sample of patients with PTSD with and without comorbid CDD. The separation of these two constructs was supported, even when measured in a highly traumatized sample and using only items from an inventory designed to assess pathological dissociation. These results replicate findings from previous investigations in patients with PTSD (Frewen et al., 2014; Frewen & Lanius, 2014b) and bipolar disorder (BPD) (Frewen et al., 2014), acutely traumatized individuals (Frewen et al., 2015), and online (Frewen et al., 2017; Tzannidakis & Frewen, 2015) and student samples (Brown & Frewen, 2017; Frewen & Lanius, 2014b). The focus on TRASC, defined as non-self-referential alterations of consciousness, therefore, in our opinion, represents a promising narrowing and delineation of the term dissociation.

The results also represent a noteworthy expansion of previous empirical support for the 4-D model in that the predictions are supported in the sample with comorbid CDD, and the finding that experiences of TRASC specifically predicted CDD membership beyond PTSD status and levels of general psychopathology. It is worth noting that although the 4-D model aims to describe pathological dissociation, it does not specifically address the diagnostic criteria of CDD (e.g. amnesia and identity alterations). As such, the items used to operationalize TRASC in this study do not measure presumed core symptoms of CDD, such as significant time loss, dissociative fugue or the presence of alters/parts of the personality. As previously noted, the 4-D model holds that experiences of TRASC do not occur exclusively within the context of identity fragmentation. This is evident in the present study, as PTSD patients without CDD comorbidity also endorsed having experiences of TRASC, albeit to a lesser degree than those with CDD comorbidity. This supports the 4-D model as a framework for understanding dissociation across different clinical presentations with different levels of psychopathology and trauma exposure.

We did not find support for the prediction that experiences of TRASC would be less intercorrelated than experiences of NWC, replicating the findings from a previous investigation in patients with BPD (Frewen et al., 2014), but contrasting with findings from samples with less pronounced psychopathology. This might point to dissociation forming a more unidimensional structure, across the four dimensions, in more clinically severe and trauma-affected samples. It is conceivable that experiences of TRASC are experienced as isolated or transient phenomena for less severely traumatized individuals, but are more pervasive across the dimensions of conscious awareness in those affected by severe childhood trauma. People with DID for instance, with distinct dissociative identity states, can typically experience a full range of dissociative phenomena simultaneously. However, the original prediction of the 4-D model regarding symptom intercorrelations refers to state-dependent assessment, that is, as taking place during discrete moments in time, which remains to be investigated.

As in previous investigations of the model in trauma-affected samples, the relationship between dissociation and trauma history was more complex than predicted, with only sexual abuse emerging as a consistent correlate of TRASC. This is in line with a recent meta-analytic review (Vonderlin et al., 2018) that also found CSA to be especially strongly related to dissociation, when compared to neglect and emotional abuse. CSA has also been identified as a potent risk factor for auditory hallucinations (Bentall et al., 2014), which are conceptualized as a symptom of TRASC in the 4-D model. Furthermore, higher reporting of childhood emotional neglect emerged as the only significant difference between patients with and without CDD comorbidity. Although physical abuse and sexual abuse are mostly hypothesized as etiological factors in the development of CDD (Brand & Frewen, 2017; Dalenberg et al., 2012), this may point to a role of experiences of emotional neglect in the development of CDD comorbidity. For example, DePrince, Huntjens, and Dorahy (2015) found alienating appraisals (i.e. ‘I am disconnected from people’, ‘ I feel lonely’) to distinguish patients with DID from those with PTSD, perhaps reflecting disturbances in the sense of self and others related to experiences of neglect.

It should be noted as a limitation that information on trauma history in this study was based on retrospective reporting, so possible recall bias may influence group differences. The measurement used did not provide detailed information related to the abuse, such as age of onset, number of events or relation to perpetrator. Also, as childhood abuse was an inclusion criterion for the study, the general level of abuse reported was very high. This makes it difficult to differentiate between groups and identify strong effects.

The implications drawn from this study must be further interpreted with caution owing to several other limitations. As in most previous investigations of the 4-D model, the operationalization of the constructs was based on a measure that was not originally designed to capture this specific model. Therefore, some constructs were less well represented in the MID and based on few or single items. The study was also based entirely on self-reports of dissociative symptoms, measured at a single time-point, and thus future research should examine the possibility of using observer-based data and repeated measurements. In addition, the sample in this investigation consisted entirely of help-seeking patients with PTSD and histories of childhood abuse, with the large majority being female. Generalizing the findings to men and less trauma-affected populations should be done cautiously. Finally, the study did not differentiate between PTSD patients with and without a dissociative subtype (Lanius et al., 2012, 2010). The dissociative subtype has been shown to be especially prevalent in PTSD patients with histories of childhood abuse, and it is therefore probable that a substantial proportion of patients in this sample would fulfil criteria for DPTSD. Future research should differentiate between PTSD and DPTSD patients, as this subgroup will probably confound results.

In spite of these limitations, this study expands on the empirical support for the 4-D model as a promising theory for understanding trauma-related reactions and disorders. Lanius (2015) further outlines how the model could guide treatment for trauma-affected patients, as TRASC of different dimensions of consciousness may require different treatments, something that future clinical studies could investigate. Studies on the impact of dissociation on treatment show inconsistent results, with some studies finding dissociation to predict poorer response (Bae, Kim, & Park, 2016; Kleindienst et al., 2016) while others fail to find such an effect (Minnen & Harned, 2012; Zoet, Wagenmans, van Minnen, & de Jongh, 2018). Heterogeneity in how dissociation is measured and conceptualized may influence the results, making it important to investigate whether experiences of TRASC affect treatment outcome; such an evaluation is planned for the present randomized controlled trial. Finally, the 4-D model can inform the limited literature on treatment of CDDs. Although psychotherapeutic treatment of these disorders has been shown to be beneficial (Brand et al., 2012), little is known about specific interventions and change mechanisms. Interventions aimed at symptoms of TRASC, as well as the proposed underlying non-self referential processing, may have promise for this patient group.
