# Supplementary material for: Trauma-related altered states of consciousness in post-traumatic stress disorder patients with or without comorbid dissociative disorders
Source: Eur J Psychotraumatol. 2018 Nov 14;9(1):1544025. doi: 10.1080/20008198.2018.1544025 (PMC6237167; doi:10.1080/20008198.2018.1544025)
Supplement: Supplemental Material [file ZEPT_A_1544025_SM0176.docx]

Supplementary material

“Trauma Related Altered States of Consciousness (TRASC) in PTSD patients with or without comorbid Dissociative Disorders”

**Hypothesis 3.**

**Repeated Measures ANOVA - Whole sample**

| **Within Subjects Effects** | | | | | | | | | | | | | | | | |  |
| --- | --- | --- | --- | --- | --- | --- | --- | --- | --- | --- | --- | --- | --- | --- | --- | --- | --- |
|  | | | **Sum of Squares** | | **df** | | **Mean Square** | | | | **F** | | **p** | | **η²** | |  |
| Form |  | | 2083.7 |  | 1 |  | 2083.745 | | |  | 397.99 |  | < .001 |  | 0.747 |  |  |
| Residual |  | | 706.8 |  | 135 |  | 5.236 | | |  |  |  |  |  |  |  |  |
| Dimension |  | | 413.0 |  | 3 |  | 137.677 | | |  | 37.00 |  | < .001 |  | 0.215 |  |  |
| Residual |  | | 1506.8 |  | 405 |  | 3.721 | | |  |  |  |  |  |  |  |  |
| Form ✻ Dimension |  | | 247.8 |  | 3 |  | 82.598 | | |  | 27.36 |  | < .001 |  | 0.169 |  |  |
| Residual |  | | 1222.4 |  | 405 |  | 3.018 | | |  |  |  |  |  |  |  |  |
|  | | | | | | | | | | | | | | | | |  |
| *Note.*  Type III Sum of Squares | | | | | | | | | | | | | | | | |  |
|  | | | | | | | | | | | | | | | | |  |
| **Post Hoc Tests** | | | | | | | | | | | | | | | | | |
|  | |  | |  | | | | **t** | | **df** | | **p** | | **Cohen's d** | | | |
| NWC_Time |  | - |  | TRASC_Time | | |  | 13.530 |  | 139 |  | < .001 |  | 1.143 | | |  |
| NWC_Thought |  | - |  | TRASC_Thought | | |  | 13.139 |  | 141 |  | < .001 |  | 1.103 | | |  |
| NWC_Body |  | - |  | TRASC_Body | | |  | 15.917 |  | 136 |  | < .001 |  | 1.360 | | |  |
| NWC_Emotion |  | - |  | TRASC_Emotion | | |  | 6.483 |  | 141 |  | < .001 |  | 0.544 | | |  |
|  | | | | | | | | | | | | | | | | | |
| Note.  Student's t-test. | | | | | | | | | | | | | | | | | |

**Repeated Measures ANOVA – Only subsample with comorbid CDD**

| **Within Subjects Effects** | | | | | | | | | | | | | | | | | | | | | | |
| --- | --- | --- | --- | --- | --- | --- | --- | --- | --- | --- | --- | --- | --- | --- | --- | --- | --- | --- | --- | --- | --- | --- |
|  | | | | | **Sum of Squares** | | **df** | | **Mean Square** | | | | | | **F** | | | | **p** | | **η²** | |
| Form | | | |  | 589.44 |  | 1 |  | 589.439 | | | | |  | 118.879 | |  | | < .001 |  | 0.725 |  |
| Residual | | | |  | 223.12 |  | 45 |  | 4.958 | | | | |  |  | |  | |  |  |  |  |
| Dimension | | | |  | 97.43 |  | 3 |  | 32.476 | | | | |  | 9.344 | |  | | < .001 |  | 0.172 |  |
| Residual | | | |  | 469.20 |  | 135 |  | 3.476 | | | | |  |  | |  | |  |  |  |  |
| Form ✻ Dimension | | | |  | 75.77 |  | 3 |  | 25.257 | | | | |  | 6.403 | |  | | < .001 |  | 0.125 |  |
| Residual | | | |  | 532.52 |  | 135 |  | 3.945 | | | | |  |  | |  | |  |  |  |  |
|  | | | | | | | | | | | | | | | | | | | | | | |
|  | | | | | | | | | | | | | | | | | | | | | | |
| **Post Hoc Tests** | | | | | | | | | | | | | | | | | |  |  |  |  |  |
|  | |  | |  | | | **t** | | | **df** | | **p** | | **Cohen's d** | | | |  |  |  |  |  |
| NWC_Time |  | - |  | TRASC_Time | |  | 6.614 | |  | 45 |  | < .001 |  | 0.975 | |  | |  |  |  |  |  |
| NWC_Thought |  | - |  | TRASC_Thought | |  | 5.993 | |  | 45 |  | < .001 |  | 0.884 | |  | |  |  |  |  |  |
| NWC_Body |  | - |  | TRASC_Body | |  | 8.757 | |  | 45 |  | < .001 |  | 1.291 | |  | |  |  |  |  |  |
| NWC_Emotion |  | - |  | TRASC_Emotion | |  | 2.572 | |  | 45 |  | 0.013 |  | 0.379 | |  | |  |  |  |  |  |
|  | | | | | | | | | | | | | | | | | |  |  |  |  |  |

**Repeated Measures ANOVA – Only subsample without comorbid CDD**

| **Within Subjects Effects** | | | | | | | | | | | | | | |
| --- | --- | --- | --- | --- | --- | --- | --- | --- | --- | --- | --- | --- | --- | --- |
|  | | **Sum of Squares** | | **df** | | **Mean Square** | | **F** | | **p** | | | **η²** | |
| Form |  | 1502.1 |  | 1 |  | 1502.091 |  | 280.90 |  | < .001 | |  | 0.759 |  |
| Residual |  | 475.9 |  | 89 |  | 5.347 |  |  |  |  | |  |  |  |
| Dimension |  | 324.5 |  | 3 |  | 108.164 |  | 28.07 |  | < .001 | |  | 0.240 |  |
| Residual |  | 1028.7 |  | 267 |  | 3.853 |  |  |  |  | |  |  |  |
| Form ✻ Dimension |  | 173.4 | ᵃ | 3 | ᵃ | 57.789 | ᵃ | 22.41 | ᵃ | < .001 | | ᵃ | 0.201 |  |
| Residual |  | 688.6 |  | 267 |  | 2.579 |  |  |  |  | |  |  |  |
|  | | | | | | | | | | | | | | |
|  | | | | | | | | | | | | | | |
|  | | | | | | | | | | | | | | |
| \| **Post Hoc Tests** \| \| \| \| \| \| \| \| \| \| \| \| \| \| \| \| \| --- \| --- \| --- \| --- \| --- \| --- \| --- \| --- \| --- \| --- \| --- \| --- \| --- \| --- \| --- \| --- \| \|  \| \|  \| \|  \| **t** \| \| **df** \| \| **p** \| \| \| **Cohen's d** \| \| \| \| \| NWC_Time \|  \| - \|  \| TRASC_Time \|  \| 11.983 \|  \| 93 \| \|  \| < .001 \| \|  \| 1.236 \|  \| \| \| NWC_Thought \|  \| - \|  \| TRASC_Thought \|  \| 12.009 \|  \| 95 \| \|  \| < .001 \| \|  \| 1.226 \|  \| \| \| NWC_Body \|  \| - \|  \| TRASC_Body \|  \| 13.278 \|  \| 90 \| \|  \| < .001 \| \|  \| 1.392 \|  \| \| \| NWC_Emotion \|  \| - \|  \| TRASC_Emotion \|  \| 6.375 \|  \| 95 \| \|  \| < .001 \| \|  \| 0.651 \|  \| \| \|  \| \| \| \| \| \| \| \| \| \| \| \| \| \| \| \| \|  \| \| \| \| \| \| \| \| \| \| \| \| \| \| \| \| | | | | | | | | | | |  |  |  |  |

**Hypothesis 5**

**Regression Analyses for TRASC and NWC scores prediction of DES and SDQ. Only sample with – Only subsample with comorbid CDD**

|  | **DES** | | | **SDQ** | | |
| --- | --- | --- | --- | --- | --- | --- |
| **Variable** | *B* | **SE B** | *β* | *B* | **SE B** | *β* |
| TRASC | 6.372 | 1.428 | .706** | 5.961 | 0.855 | 1.077** |
| NWC | 1.287 | 1.534 | .133 | -2.393 | 0.918 | -.403* |
| R^2^ | .656  29.53** | | | .678  31.63** | | |
| F |  |  |  |  |  |  |

*p < .05. **p < .01.

**Regression Analyses for TRASC and NWC scores prediction DES and SDQ. Only sample with – Only subsample without comorbid CDD**

|  | **DES** | | | **SDQ** | | |
| --- | --- | --- | --- | --- | --- | --- |
| **Variable** | *B* | **SE B** | *β* | *B* | **SE B** | *β* |
| TRASC | 7.448 | 2.337 | .715** | 4.953 | 0.634 | .865** |
| NWC | 1.306 | 0.931 | .186* | -0.518 | 0.428 | -.134 |
| R^2^ | .736  94.84** | | | .601  50.36** | | |
| F |  |  |  |  |  |  |

*p < .05. **p < .01.
